# Facile one-pot hydrothermal synthesis of a zinc oxide/curcumin nanocomposite with enhanced toxic activity against breast cancer cells

**DOI:** 10.1039/d3ra05176e

**Published:** 2023-09-11

**Authors:** Lorenzo Francesco Madeo, Christine Schirmer, Giuseppe Cirillo, Samuel Froeschke, Martin Hantusch, Manuela Curcio, Fiore Pasquale Nicoletta, Bernd Büchner, Michael Mertig, Silke Hampel

**Affiliations:** a Leibniz Institute for Solid State and Materials Research Dresden Dresden 01069 Germany l.f.madeo@ifw-dresden.de +49 3514659883; b Kurt-Schwabe-Institut für Mess- und Sensortechnik Meinsberg e.V. Kurt-Schwabe-Straße 4 Waldheim 04736 Germany; c Department of Pharmacy, Health and Nutritional Sciences, University of Calabria Rende 87036 CS Italy; d Institute of Solid State and Materials Physics, Technische Universität Dresden Dresden 01062 Germany; e Institute of Physical Chemistry, Technische Universität Dresden Dresden 01062 Germany

## Abstract

Zinc oxide/Curcumin (Zn(CUR)O) nanocomposites were prepared *via* hydrothermal treatment of Zn(NO_3_)_2_ in the presence of hexamethylenetetramine as a stabilizing agent and CUR as a bioactive element. Three ZnO : CUR ratios were investigated, namely 57 : 43 (Zn(CUR)O-A), 60 : 40 (Zn(CUR)O-B) and 81 : 19 (Zn(CUR)O-C), as assessed by thermogravimetric analyses, with an average hydrodynamic diameter of nanoaggregates in the range of 223 to 361 nm. The interaction of CUR with ZnO *via* hydroxyl and ketoenol groups (as proved by X-ray photoelectron spectroscopy analyses) was found to significantly modify the key properties of ZnO nanoparticles with the obtainment of a bilobed shape (as shown by scanning electron microscopy), and influenced the growth process of the composite nanoparticles as indicated by the varying particle sizes determined by powder X-ray diffraction. The efficacy of Zn(CUR)O as anticancer agents was evaluated on MCF-7 and MDA-MB-231 cancer cells, obtaining a synergistic activity with a cell viability depending on the CUR amount within the nanocomposite. Finally, the determination of reactive oxygen species production in the presence of Zn(CUR)O was used as a preliminary evaluation of the mechanism of action of the nanocomposites.

## Abstract 2

A novel straightforward hydrothermal method was used to combine ZnO and CUR in a stable nanocomposite with remarkable anticancer properties

## Graphical abstract

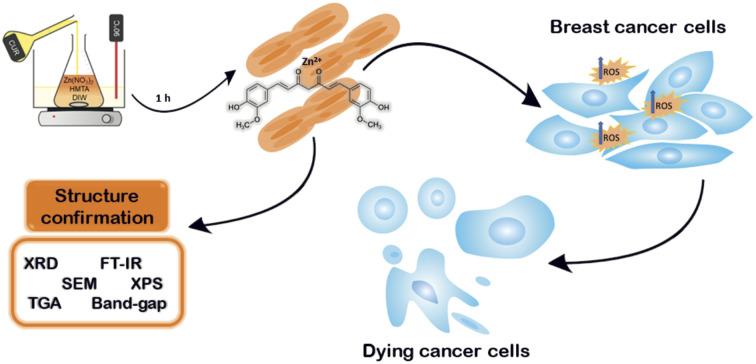

## 1 Introduction

Accounting for a quarter of all cancer cases in women, breast cancer is the most commonly diagnosed cancer worldwide.^1^ Surgery and chemotherapy are the most common treatments, with other options including hormonal therapy, radiotherapy and targeted therapy.^2^ In particular, chemotherapy can be used alone or in combination with other treatments, depending on the type of cancer and how far it has spread.^3^ Especially in the early stage of triple-negative breast cancer (TNBC), chemotherapy may play a key role as neoadjuvant, maintaining or reducing the tumour size before surgical removal.^4^ However, it has been reported that increased drug efflux pumps, decreased intracellular drug concentrations, and decreased drug uptake are associated with the development of drug resistance in TNBC.^5^ In addition, unpleasant side effects, which increase with the frequency of drug administration, and frequent relapses are proved to be the main disadvantages of traditional chemotherapy.^3^ Such a scenario has motivated researchers to develop new approaches based on nanotechnology. In this context, nanomedicine offers valuable tools for therapeutic strategies against challenging cancers. Nanoparticles (NPs) can be used as drug delivery platforms able to specifically target cancer cells by virtue of their tailored physicochemical and biological properties.^6^ Compared to conventional drug formulations, NPs are able to overcome pharmacokinetic limitations associated with low solubility, inability to penetrate tumours, and damage to the immune system and other organs.^7^ Nanosystems used for cancer treatment include liposomes,^8^ polymeric micelles,^9^ nanospheres, DNA origami-based nanocarriers,^10^ quantum dots,^11^ and carbon-^12^ and metal-^13^ based nanoparticles.

Zinc oxide nanoparticles (ZnO NPs) showed remarkable properties as an emerging semiconductor material with a wide range of applications, including biomedicine, due to their biocompatible nature.^6^ Zinc is a trace mineral, essential for the activation of several enzymes with a role in cell growth, protein and DNA synthesis, and tissue healing.^14^ Likewise, ZnO NPs are biocompatible towards mammalian cells due to their low dissolution rate under physiological conditions (pH = 7.4), while they become toxic at slightly acidic pH as consequence of rapid release of Zn^2+^ ions, causing oxidative stress and cell damage.^15^ Since acidification of the extracellular medium is a hallmark of the tumour environment,^16^ this feature plays a key role in selective cancer toxicity. ZnO NPs have been employed as both drug carrier and active toxic agent towards TNBC cell lines,^17^ with morphology and size playing a key role in their efficacy. ZnO NPs have been synthesized with various methods, such as thermal decomposition, sol–gel routes, hydrothermal methods, ultrasonication and green synthesis.^18^ The resulting particles can be functionalised with polymers to enhance hydrophilicity and drug encapsulation efficiency, and displayed a variety of morphologies, including spheres, dumbbells, rods, flowers, tubes, and hexagonal prisms.^19^

Alongside conventional chemotherapeutic molecules, a growing number of naturally occurring molecules have been identified as potential candidates for cancer treatment. Among these, curcumin (CUR), a diferuloylmethane constituent of the yellow pigments isolated from *Curcuma longa*, has been extensively investigated for its potent anti-proliferative effects due to its ability to interfere with different cellular pathways involved in carcinogenesis.^20,21^ CUR has been tested against a variety of cancers, including prostate cancer,^22^ squamous cell carcinoma,^23^ colorectal cancer,^24^ glioblastoma,^25^ and breast cancer.^26^ CUR has demonstrated anti-proliferative activity on cell cycle regulatory proteins, matrix metalloproteinases and NF-κB in the TNBC cell line MDA-MB-231,^27^ as well as inhibitory activity on the inflammatory cytokines CXCL1/2, resulting in inhibition of the expression of a number of metastasis-promoting genes.^28^ While various phase I clinical trials have reported that CUR is well tolerated even at high doses, its reduced bioavailability has been the major drawback for its therapeutic use.^29^ Researchers have therefore attempted to overcome these obstacles through functionalisation, chemical modification, nanoparticles formulation, and the use of adjuvants to interfere with CUR metabolism.^30^ Metal oxide particles, such as ZnO NPs, can provide a suitable delivery system to overcome the limitations of CUR as a pure therapeutic agent,^31^ and at the same time exert their own biological activity, resulting in a synergistic effect.

In this study, ZnO and CUR have been combined in a stable composite (Zn(CUR)O), through a one-pot, straightforward hydrothermal synthesis. Zn(NO_3_)_2_ has been used as source of Zn^2+^ ions for ZnO NPs, while hexamethylenetetramine (HMTA) in aqueous solution has provided a weak basic environment and has stabilized Zn^2+^ during the reaction.^32^ The conjugation with three different concentrations of CUR affected the ZnO NPs morphology and crystallite size, shifting from hexagonal prisms to roughly dumbbell-shaped aggregates of nanosized crystals. Zn(CUR)O composites were tested in terms of toxic effect on both MCF-7 and MDA-MB-231 breast cancer cells, and the production of reactive oxygen species (ROS) over time was investigated as the main biological mechanism.

## 2 Experimental

### 2.1. Synthesis of ZnO NPs and Zn(CUR)O composite

Zn(CUR)O nanocomposites were synthesised according to the conventional growth of ZnO NPs from aqueous solution.^32,33^ In contrast to this method, no substrate was used. Zn(NO_3_)_2_ × 6 H_2_O (0.025 M) and hexamethylenetetramine (HMTA, C_6_H_12_N_4_, 0.0125 M) were dissolved in deionised water (DIW), together with three different concentrations of CUR, previously dissolved in ethanol. According to the concentration of CUR used (5.4 × 10^−4^ M, 2.7 × 10^−4^ M and 9.05 × 10^−5^ M), the products were labelled Zn(CUR)O-A, Zn(CUR)O-B, and Zn(CUR)O-C, respectively. The reaction mixture was stirred at 90 °C for 60 minutes in a thermostatically controlled oil bath. The final product was collected and purified by centrifugation in DIW for two times (Allegra 64 R, Beckman Coulter, Brea, CA, USA) and subsequently in acetone to remove excess CUR. The complete removal of unreacted CUR was checked by high pressure liquid chromatography (HPLC) analysis of the dialysis medium using a Jasco PU-2089 Plus liquid chromatograph equipped with a Rheodyne 7725i injector (fitted with a 20 mL loop), a Jasco UV-2075 HPLC detector operating at 420 nm, and a Jasco-Borwin integrator (Jasco Europe s.r.l., Milan, Italy). The stationary phase consisted of a Tracer Excel 120 ODS-A column, particle size 5 mm, 15 × 0.4 cm (Barcelona, Spain). Since no interfering compounds were present in the reaction feed, methanol at a flow rate of 1.0 mL min^−1^ was used as the mobile phase.^34^ The limits of detection and quantification were 0.05 and 0.50 μg mL^−1^, respectively, with a linearity assessed by analysing a series of CUR standard solutions within the concentration range of 0.50 to 50.0 μg mL^−1^, in agreement with the available literature protocols.^35,36^ The intra-day precision (*p* < 0.05, student's *t*-test) was assessed by injecting CUR solutions in triplicate at 0.50, 25.0 and 50.0 μg mL^−1^, corresponding to low, average, and high concentrations in the calibration curve range. The purified powder was collected by vacuum filtration and dried at 60 °C overnight. Depending on the amount of CUR used in the synthesis, the products obtained were orange in colour, with different tonalities. ZnO NPs were synthesised under the same conditions in the absence of CUR. All chemicals were from Merck KGaA, Darmstadt, Germany.

### 2.2. Characterization procedures

Powder X-ray diffraction (pXRD) was performed with Cu K_α1_ radiation (*λ*_Kα1_ = 1.54060 Å) in transmission geometry (STOE STADI-P, Ge (111) primary beam monochromator, Mythen 1K detector, 0.015° step size, 191 mm camera distance). The crystallite domain sizes were calculated from the diffraction pattern with the software GSAS-II version 5311, using a uniaxial domain size model with 001 as the unique axis.^37^

Scanning electron microscope (SEM) images of the samples were obtained using a NOVA NanoSEM 200 [5–15 kV] (Thermo Fisher Scientific, Hillsboro, OR, USA) by deposition of previously water-dispersed samples on Si/SiO_2_ substrates (Plano GmbH, Wetzlar, Germany).

Dynamic light scattering (DLS) analyses were performed with a 90 Plus Particle Size Analyzer (Brookhaven Instruments Corp, Holtsville, NY, USA) at 25.0 ± 0.1 °C with a laser operating at 658 nm while measuring the autocorrelation function at 90°.

Fourier transform infrared spectroscopy (FTIR) spectra were recorded using a Vertex80v (BRUKER Optic GmbH, Karlsruhe, Germany) with KBr pellet technique, with 250 scans performed for each spectrum.

Photoelectron spectroscopy (XPS) was carried out using a PerkinElmer PHI-5600 spectrometer equipped with a 150 mm hemispherical analyser (PerkinElmer, Inc., Waltham, MA, USA). A non-monochromatic Mg-Kα X-source (250 W) was chosen and the data is satellite corrected. The powder samples required a special geometry which leads to an electron emission angle of 45° relative to the analyser. A neutraliser is used to avoid charging effects and the binding energy scale is corrected based on the shift of C 1s graphitic carbon at 284.8 eV for each spectrum. High resolution spectra are carried out with a 29.35 eV pass energy.

Thermogravimetric analysis (TGA) was performed on an SDT Q600 (TA Instruments, Hüllhorst, Germany) under a nitrogen atmosphere with the following conditions: 2 mg initial sample weight, 10 mL min^−1^ N_2_ flow, 25–800 °C temperature range, and 10 °C min^−1^ heating rate.

For diffuse reflection spectroscopy, samples were previously pressed onto BaSO_4_ pellets and measured with a Shimadzu UV-3101PC Spectrometer with an attached multi-purpose large-sample compartment MPC-3100.

### 2.3. Cell culture and sample preparation

Human breast cancer cell lines MCF-7 and MDA-MB-231 were obtained from the DSMZ-German Collection of Microorganisms and Cell Cultures GmbH and cultured in DMEM supplemented with 10% fetal bovine serum, 100 U/ml penicillin, 100 μg mL^−1^ streptomycin and 0.25 μg mL^−1^ amphotericin B (all purchased from PAN-Biotech GmbH, Aidenbach, Germany). For the MCF-7 cells, 10 μg mL^−1^ human recombinant insulin (PAN-Biotech GmbH, Aidenbach, Germany) were added to the medium. Cells were grown as monolayers in tissue culture flasks at 37 °C in a humidified atmosphere of 5% CO_2_. Cell cultures were free of mycoplasma as determined with MycoStrip Mycoplasma detection kit (InvivoGen, Toulouse, France).

For determining cytotoxic activity and ROS-inducing potential of the synthesised Zn(CUR)O composites, ZnO NPs and pure CUR, samples were pre-diluted in cell culture grade DMSO (PAN Biotech, Aidenbach, Germany) and afterwards dissolved in media without phenol red without or with serum.

Cells were seeded in 96-well plates at 5 × 10^5^ cells per well (MCF-7) or 4 × 10^5^ cells per well (MDA-MB-231), grown to 70–80% confluence and treated with the composites as well as controls for 48 h.

Microscopy images of the cells were taken after treatment using an Axio observer-Z1 microscope (Carl Zeiss Microimaging, Jena, Germany) equipped with an Evolve-EM 512 camera (Photometrics, Tucson, AZ, USA).

### 2.4. Cell viability assay

WST-1 assay (4-[3-(4-Iodophenyl)-2-(4-nitro-phenyl)-2H-5-tetrazolio]-1,3-benzene sulfonate; Roche Diagnostics GmbH, Mannheim, Germany) is a colorimetric assay that measures mitochondrial activity thereby reflecting cellular viability. The assay was performed as described previously.^38^ To this aim, half of the treated wells were incubated with 0.2% Triton X-100 (Merck KGaA, Darmstadt, Germany) to kill cells, while the other half was cultured with medium. WST-1 was then added to all of the wells and absorbance (Abs) was measured after 3.5 h with a microplate reader (BioTek Synergy H1, Agilent Technologies, Santa Clara, CA, USA) at 480 nm, and at 630 nm as a reference. The percentage of surviving cells was calculated relative to control cells treated with solvent only according to the following eqn (1):1
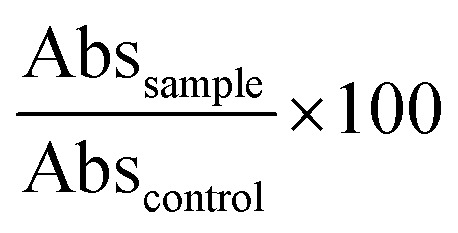
where Abs was obtained by the following eqn (2):

2 Abs = (Abs_480_ − Abs_630_)_no Trit_ − (Abs_480_ − Abs_630_)_Trit_

Therewith, possible interferences from nanoparticles with the absorbance readings and/or the WST-1 substrate are considered.

### 2.5. ROS assay

For fluorescence-based measurement of reactive oxygen species in the cancer cells, DCFDA (2′,7′ – dichlorofluorescin diacetate) cellular ROS assay kit (Abcam, Germany) was used according to manufacturer's instructions. In brief, 45 min prior to adding the samples to the cells, DCFDA was added to the wells. Fluorescence (FI) was measured with a microplate reader (BioTek Synergy H1, Agilent Technologies, Santa Clara, CA, USA) after 4 h incubation with *λ*_ex_ and *λ*_em_ = 485 and 535 nm, respectively. Relative fluorescence intensity based on the control was calculated according to eqn (3):3
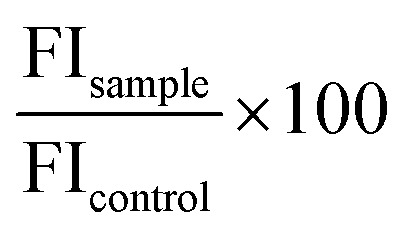


### 2.6. Statistical analyses

Biological data are presented as mean of three replicates with error bars showing standard deviations. A two-tailed *t*-test was used to test for the differences in treatments, while *p* less than 0.05 considered statistically significant.

## 3 Results and discussion

### 3.1. Synthesis and characterisation of Zn(CUR)O composite

**Fig. 1:**
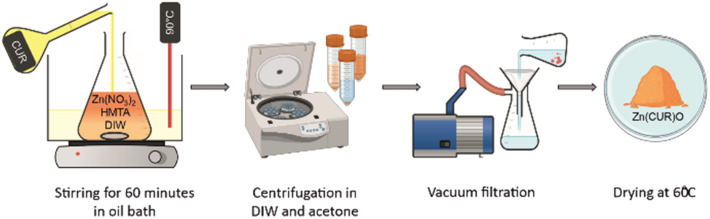
Schematic representation of the applied synthetic procedure.

Zn(CUR)O nanocomposites were obtained by a simple straightforward procedure, without the use of toxic solvents and at relatively low temperatures (Fig. 1).

For ZnO NPs, zinc nitrate provides Zn^2+^ ions, while HMTA is supposed to play multiple roles in the reaction. Due to its non-ionic cyclic tertiary amine structure, it is thought to act as bidentate Lewis base, coordinating two Zn^2+^ ions.^39^ HMTA hydrolyses in water, releasing NH_3_ and providing a weak basic environment for Zn^2+^ to form Zn(OH)_2_, which eventually dehydrates to ZnO. The series of reactions can be summarised as follows (eqn (4)):4
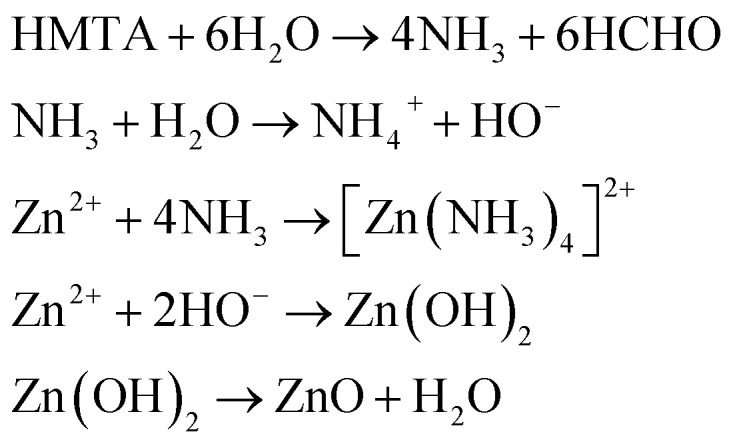


The ZnO growth mediated by HMTA aqueous solution was preferred over growth in strong alkaline solutions in order to partially prevent the CUR degradation at extremely high pH.^40^ The addition of pure CUR at three different concentrations to the reaction environment conferred an orange colour to the previously white ZnO powder, obtaining Zn(CUR)O-A, Zn(CUR)O-B and ZnO(CUR)O-C. Several different concentrations of CUR were tested beforehand. The lowest concentration chosen resulted to be the minimum for obtaining a significant modification of the ZnO properties. On the other hand, further increases in CUR concentration above the highest used in this work did not show substantial differences in terms of composite composition and toxic activity towards cancer cells (see below).

The repeated centrifugation steps with acetone washed away the excess of CUR that was adsorbed on the ZnO surface.

The persistent orange colour of the powder was an indication of a significant chemical bonding between the CUR and the ZnO, forming a composite.

**Fig. 2:**
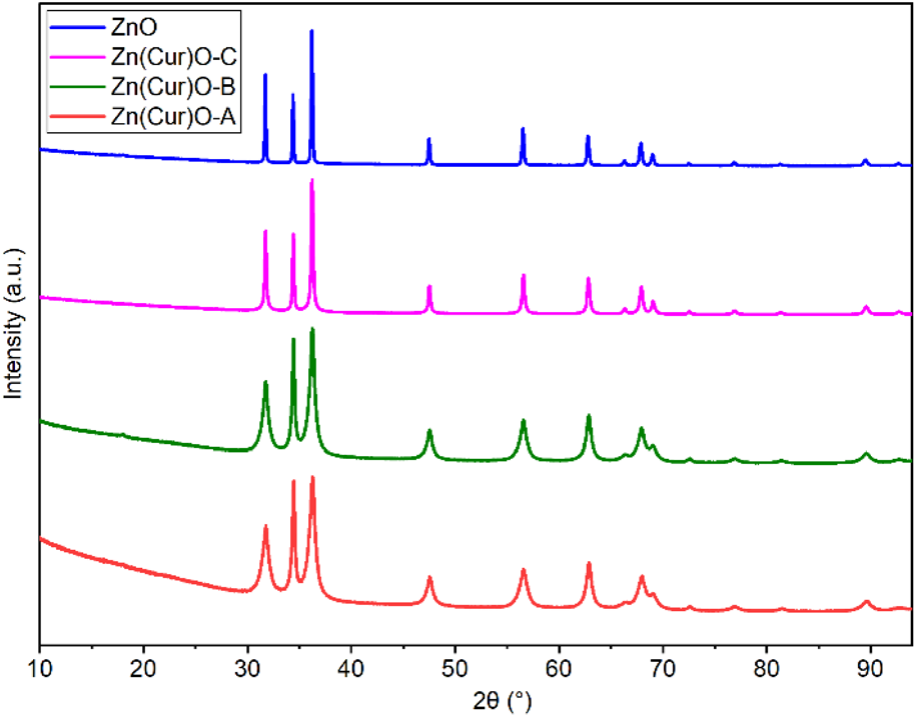
pXRD spectra of ZnO and Zn(CUR)O composites.

In order to confirm the purity of the samples, investigate their crystal structure, and determine the crystallite size, pXRD was performed on pure ZnO and Zn(CUR)O composites (Fig. 2).

The presence of all the ZnO hexagonal phase diffraction peaks reported in the literature confirmed the hexagonal ZnO wurtzite structure of all the samples analysed. The absence of any additional peaks demonstrated the crystalline purity of the samples. Furthermore, no CUR peaks were observed, indicating the amorphous nature of curcumin in the composites.

At the same time, a progressive broadening of the peaks was observed as the CUR content increased from pure ZnO NPs to Zn(CUR)O-A. This was associated with a decrease in crystallite size to nanoscale dimensions.^41^ By the Scherrer equation, the equatorial crystallite sizes were calculated to be 104.6 ± 0.7 nm for ZnO, 12.6 ± 0.1 nm for Zn(CUR)O-A, 15.6 ± 0.1 nm for Zn(CUR)O-B and 47.1 ± 0.2 nm for Zn(CUR)O-C, while values of 106.1 ± 1.2 nm for ZnO, 28.4 ± 0.3 nm for Zn(CUR)O-A, 33.3 ± 0.3 nm for Zn(CUR)O-B and 66.7 ± 0.6 nm for Zn(CUR)O-C were obtained for the axial size. The results suggest that CUR in amorphous form interacts with crystalline ZnO without affecting its crystalline structure, but hinders its growth by capping the outer faces, slowing down the overall growth process and ultimately reducing the crystallite sizes.

**Fig. 3:**
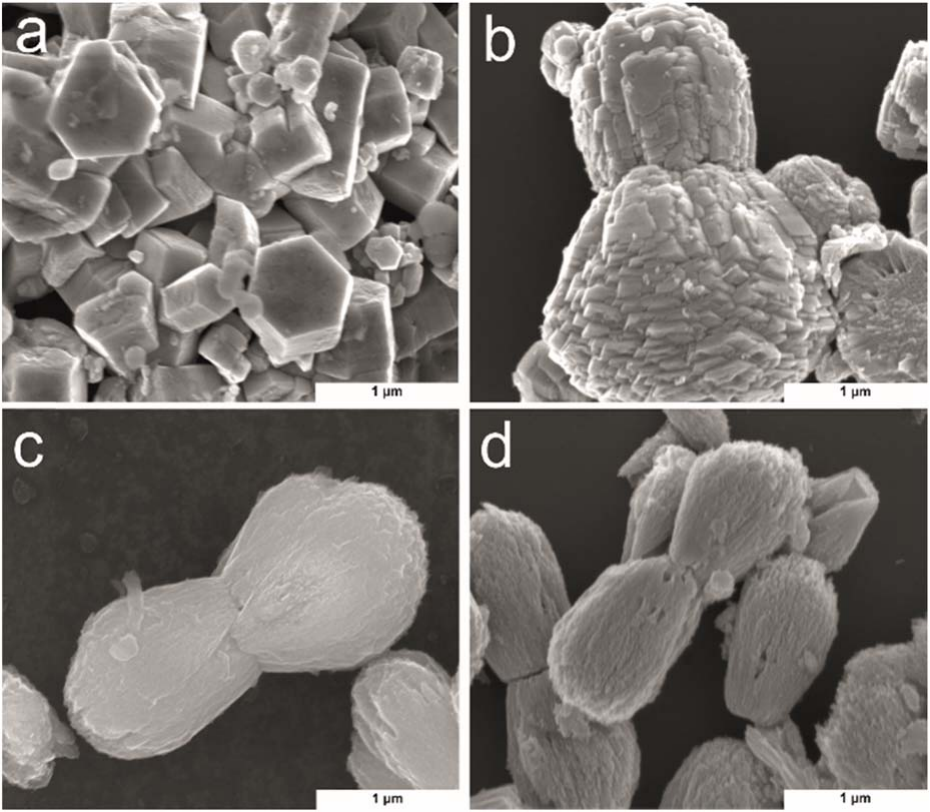
SEM images of (a) ZnO; (b) Zn(CUR)O-C; (c) Zn(CUR)O-B; (d) Zn(CUR)O-A.

SEM was used to investigate the morphology of the Zn(CUR)O with different CUR content, in comparison to the pure ZnO NPs (Fig. 3). To get a better image of the surface morphology of the particles, larger particles were considered. ZnO exhibited a typical growth morphology of hexagonal pillars for ZnO.^42^ In contrast with the methods involving substrate for crystal nucleation, here the particles have wider dimensions in all three dimensions. In presence of CUR, ZnO crystal agglomerates in bilobed shaped form (Fig. 3b–d). At low concentrations of CUR (Zn(CUR)O-C), the smooth surface of ZnO is still visible, while less defined surface shapes were observed at higher concentrations of CUR (Zn(CUR)O-A and B).

To assess the applicability of the synthesized nanocomposites in biological systems, their sizes and the size distributions were measured in DIW by DLS analyses.

The hydrodynamic diameter (HD) was clearly affected by the presence of CUR in the composite. Zn(CUR)O-A was found to have the lowest HD (223 nm, Polidispersivity index – PDI = 0.14), with an evident drop of size compared to the pure ZnO NPs (440 nm, PDI = 0.4). Intermediate values were found for Zn(CUR)O-B (245 nm, PDI = 0.32) and Zn(CUR)O-C (361 nm PDI = 0.24). These results proved the effective obtainment of nanosystems within the accepted range for biomedical applications.^43–45^

**Fig. 4:**
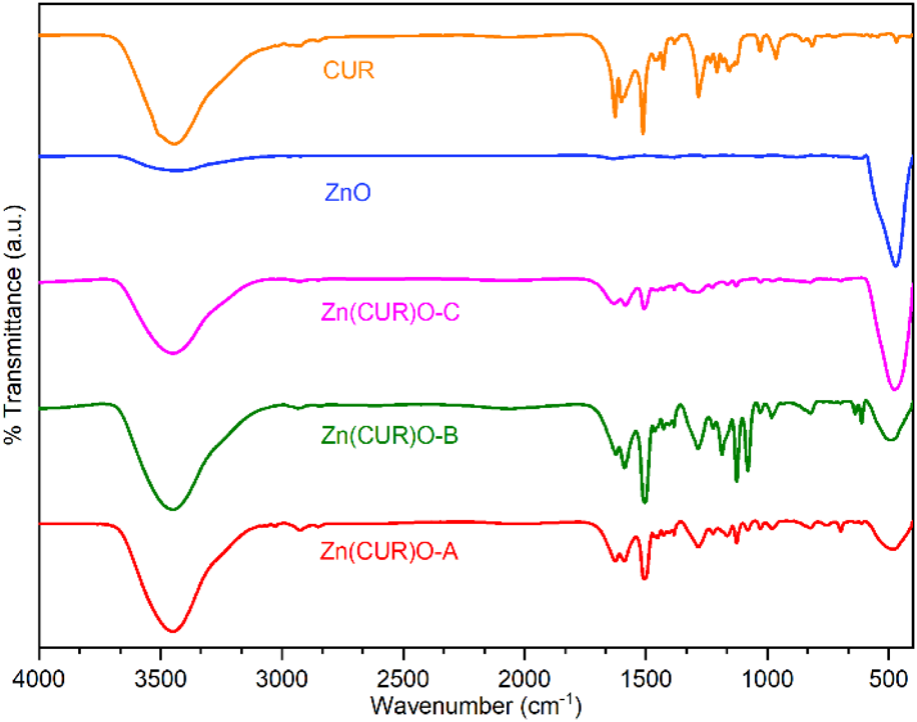
FTIR spectra of CUR, ZnO NPs and Zn(CUR)O nanocomposites.

FTIR spectra of ZnO, CUR and their composites (Zn(CUR)O-A, Zn(CUR)O-B and Zn(CUR)O-C) are shown in Fig. 4. A relevant absorption peak in the range of 400–500 cm^−1^ was observed in the spectra of pure ZnO and Zn(CUR)O composites, which was assigned to the characteristic stretching vibration of the Zn–O bond.^46^

A rounded broad peak at 3450 cm^−1^ in the spectra of pure CUR and Zn(CUR)O composites is related to the phenolic O–H stretching vibration. In the same region, a similar peak is also present to a lesser extent in the spectrum of ZnO, which is most likely due to residual hydroxyl groups on the surface of the oxide structure.

With respect to CUR, peaks were found for aromatic C=C stretching (1626 cm^−1^), benzene ring stretching (1588 cm^−1^), C=O vibrations (1510 cm^−1^), olefinic C–H bending vibrations (1427 cm^−1^) and aromatic C–O stretching vibrations (1280 cm^−1^). Spectra of Zn(CUR)O composites showed the same peaks, with slight broadening and shifts for the C=O vibration peaks (1504 cm^−1^), which can be related to the interaction of Zn^2+^ ions with keto-enolic moieties of CUR. FTIR data confirmed the presence of both CUR and ZnO in every composite. In general, Zn(CUR)O-C showed more pronounced signals related to ZnO than Zn(CUR)O-A and Zn(CUR)O-B, while more pronounced signals related to CUR were observed in Zn(CUR)O-A.

**Fig. 5:**
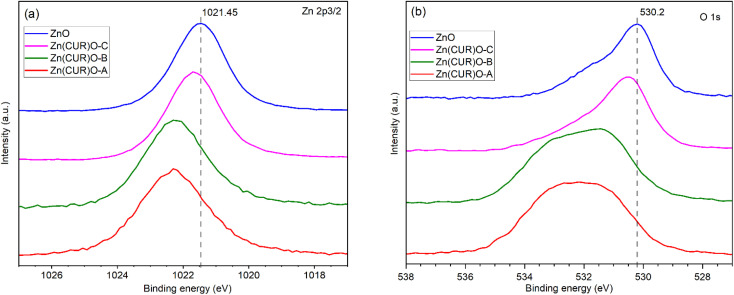
XPS analyses of ZnO NPs and Zn(CUR)O nanocomposites. (a) Zn 2p_3/2_ region; (b) O 1s region.

XPS analysis was performed to investigate the surface chemical composition of CUR, ZnO and Zn(CUR)O composites (Fig. 5). The binding energies were calibrated by taking the C 1s peak (284.8 eV) as reference. In the Zn 2p_3/2_ region (Fig. 5a), ZnO showed a peak at 1021.5 eV, related to ZnO, whose binding energy shifts are reported to be in the same range (1021 eV–1023 eV).^47^ In the Zn(CUR)O-C spectrum a slight shifting to 1021.69 eV was observed, while in the Zn(CUR)O-A and Zn(CUR)O-B spectra a further shift and an evident broadening (1022.32 eV) suggest a more inhomogeneous chemical environment around the Zn atoms. This is related to the coordination of Zn to the keto-enolic group of CUR, as reported elsewhere in the literature.^48^ In the O 1s region (Fig. 5b), the peak at 530.2 eV corresponds to the O^2−^ of the wurtzite structure of ZnO.^49^ In the spectra of Zn(CUR)O-A and Zn(CUR)O-B, very broad signals at higher binding energies can be ascribed to the presence of multiple groups, such as C=O and Zn–OH.^50^ These peaks are in good agreement with the hypothesis that Zn interacts with CUR moieties, such as hydroxyl and keto-enol groups.

**Fig. 6:**
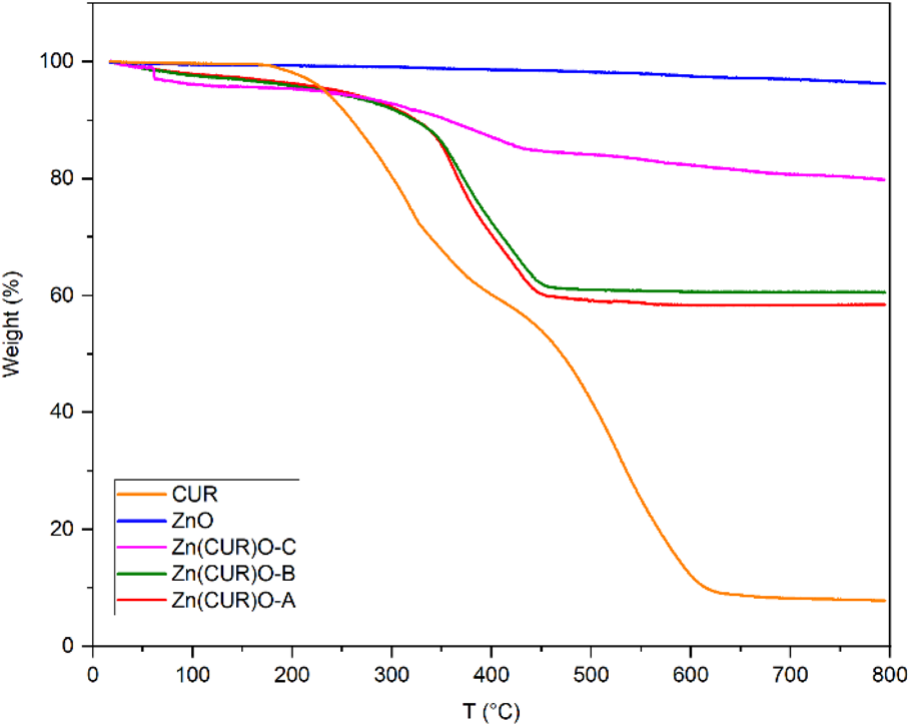
Thermogravimetric analyses of CUR, ZnO NPs and Zn(CUR)O nanocomposites.

Thermogravimetric analyses were performed to evaluate the thermal stability of the composites under analysis, compared with ZnO and CUR, and their composition ratios (Fig. 6). As expected, ZnO is thermally stable up until 800 °C, showing only a minor loss of weight (3.7%) potentially due to the thermal decomposition of residual zinc hydroxide.^51^ CUR showed multiple stages of thermal decomposition. A first weight loss starts at 170 °C, related to dehydroxylation. For the Zn(CUR)O composites this stage is shifted and less prominent, suggesting a stabilising effect mediated by Zn interaction with –OH group of CUR. At 60 °C, Zn(CUR)O-C exhibits a one-step weight loss (2%), related to the elimination of crystalline water,^52^ while Zn(CUR)O-A and Zn(CUR)O-B shows a gradual weight loss (7%) until 290 °C. The subsequent degradation stage of CUR starts at 320 °C, involving the decomposition of other groups such as benzene rings.^53^ This stage is shifted at 365 °C for the Zn(CUR)O composites. Overall, the results highlight that an increase in the ZnO content of the composites led to a minor loss in weight, indicating higher thermal stability.^54^ Since the same amount of ZnO was used for the synthesis of all the composites, by mean of the TGA weight percentage loss at 800 °C, it was possible to calculate the ZnO : CUR ratios of Zn(CUR)O composites, which resulted to be 57 : 43 for Zn(CUR)O-A, 60 : 40 for Zn(CUR)O-B and 81 : 19 for Zn(CUR)O-C.

Despite the double amount of CUR in the initial reactions of Zn(CUR)O-A compared to Zn(CUR)O-B, the two composites resulted to have similar composition ratios, further suggesting that ZnO is only capable of binding a limited amount of CUR and that increase in CUR leads to a saturation of the CUR amount of the composite particles.

**Fig. 7:**
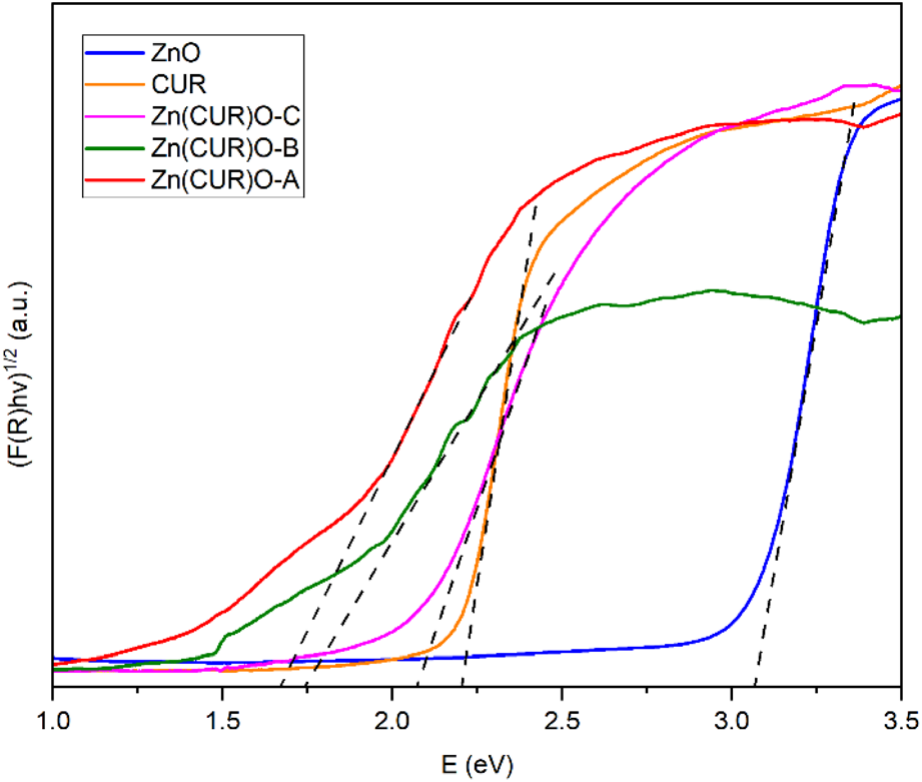
Tauc plot of the diffuse reflection spectroscopy results with applied tangents for bandgap determination of CUR, ZnO NPs and Zn(CUR)O nanocomposites.

Some previous researches raised the hypothesis that band gap shifts of metal oxides at biologically relevant energies may be related to ROS production.^55^ A previously proposed theoretical model claimed the possibility to predict the oxidative stress potential of oxide nanoparticles towards biological material, by checking if the conduction band of such oxides is superimposing on the standard redox potentials of couples active in biological media, ranging between −4.12 eV and −4.84 eV.^56^ The conduction band of ZnO lies higher with respect to the mentioned range, suggesting that its toxicity is mainly related to solubility and ion leaching.^57^ Nevertheless, it is possible to consider that reducing the band gap of ZnO may increase the chances of having band energy values comparable to the interesting range. In order to investigate whether the synthesised Zn(CUR)O composites present any band gap shift with respect to the pure ZnO, their optical band gaps were examined by diffuse reflection spectroscopy. By transforming the obtained data with the Kubelka–Munk function, they were fitted with the Tauc plot method (*γ* = 1/2 for direct band gap) as shown in Fig. 7. The optical band gap of ZnO was found to be 3.07 eV, a value in good agreement with rod-shaped nanoparticles,^58^ while for pure CUR the plot resulted in a band gap of about 2.20 eV.

For the Zn(CUR)O composite materials, the determination of the exact optical band gap is more difficult, as the spectra show additional Urbach tails, most likely as a result of the amorphous nature of the CUR part of the composite. When accounting for that, the absorption spectra and the determined optical band gaps are significantly different from those of pure CUR and ZnO and are not a simple overlapping of the two individual spectra. Hereby the spectra of Zn(CUR)O-A and Zn(CUR)O-B are again quite similar, while Zn(CUR)O-C shows a slightly higher optical band gap that is still different from the pure components. This clearly suggests an interaction between the CUR shell and the ZnO core of the composite materials, while red-shifting the band gap and adding the possibility for adsorption in the red and near infrared energy range.

### 3.2. Biological evaluation

To evaluate the efficacy of Zn(CUR)O as anticancer agents, its biological activity was compared to both pure CUR and unmodified ZnO NPs. First, MCF-7 was tested as model cell line for estrogen-responsive human breast cancer.

**Fig. 8:**
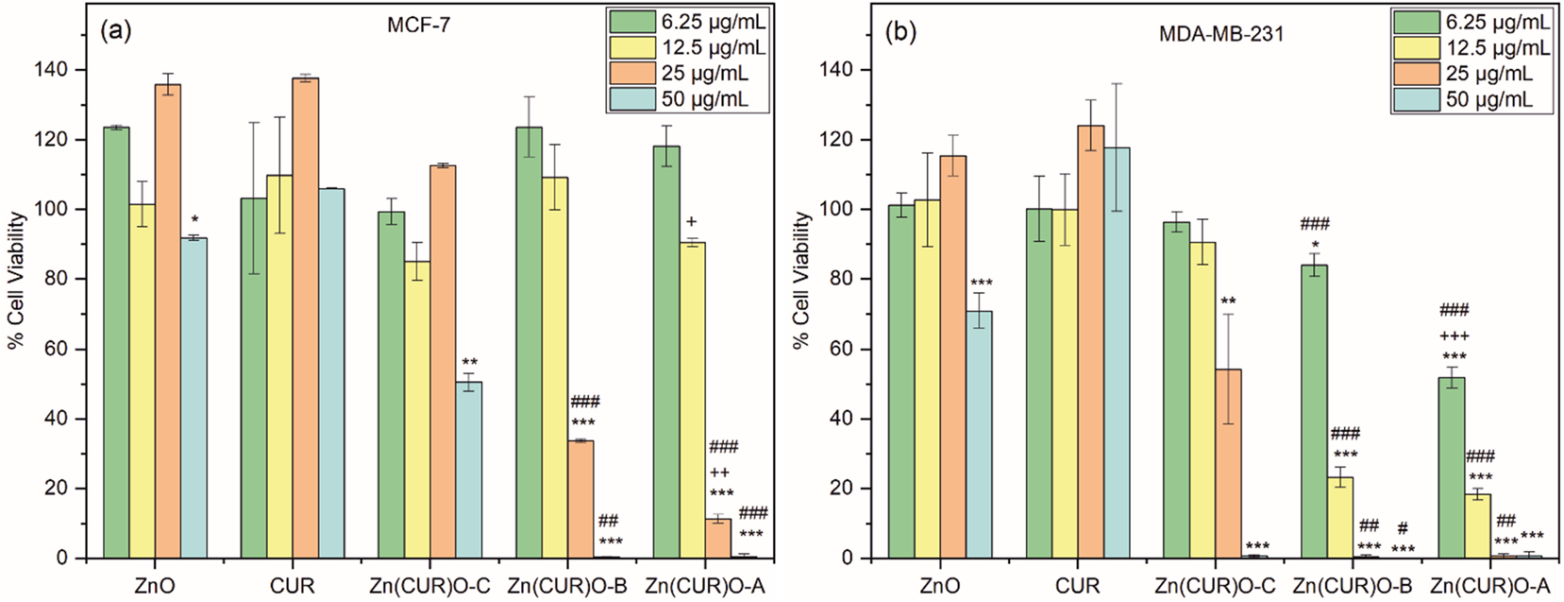
Breast cancer cells viability upon 48 h treatment with ZnO NPs, CUR and Zn(CUR)O nanocomposites in the concentration range 6.25–50 μg mL^−1^ ***/**/* *vs.* control (DMSO); ###/##/# *vs.* Zn(CUR)O-C; +++/++/+ *vs.* Zn(CUR)O-B; */#/+ *p* < 0.05; **/##/++ *p* < 0.01; ***/##/+++ *p* < 0.001.

Toxicity profiles were evaluated in terms of cell viability after 48 h incubation, testing four different concentrations for each sample (50, 25, 12.50 and 6.25 μg mL^−1^). As shown in Fig. 8a, both ZnO and CUR did not show any toxic effect over the whole range of concentrations tested, with the exception of a very small decrease of viability in cells treated with the highest concentration of ZnO. For the Zn(CUR)O composites, at the tested incubation time the toxicity was concentration dependent, while hardly any effect was measured at the concentrations of 12.50 and 6.25 μg mL^−1^ for all the three composites, Zn(CUR)O-A and Zn(CUR)O-B showed a remarkable efficacy starting from the concentration of 25 μg mL^−1^, reducing the cell viability to 11% and 34%, respectively.

At the highest concentration (50 μg mL^−1^), cells incubated with Zn(CUR)O-A and Zn(CUR)O-B showed no viability, while the survival rate of those treated with Zn(CUR)O-C was reduced to 50%. A clear role of the CUR content in the toxicity of Zn(CUR)O was demonstrated by the reported data, with a decreasing survival rate of the cancer cells associated with increasing CUR levels within the composites. According to the previous calculation of Zn : CUR ratios, the CUR contents in Zn(CUR)O-A, Zn(CUR)O-B and Zn(CUR)O-C (21.5, 20.0 and 9.5 μg mL^−1^, respectively, when the particles concentration is 50 μg mL^−1^) fall within the concentration range of pure CUR tested, which showed no effect. Therefore, a clear improvement of cytotoxic activity was achieved by a synergistic effect between ZnO and CUR in the synthesised composite.

Our data are consistent with previous research, showing that ZnO does not exert any cytotoxic activity on MCF-7 cells at the concentrations employed in our study, while it has anticancer activity towards the cells at higher concentrations.^59^

After observing a clear toxic effect of Zn(CUR)O on MCF-7 cells, the obtained composite was tested against MDA-MB-231, a highly aggressive and invasive TNBC cell line. As for the previously treated cell line, Fig. 8b shows that CUR has no toxic effect at any of the concentrations tested. ZnO NPs only showed a reduction in cell viability (71%) at the highest concentration used. Considering the composites, Zn(CUR)O-C showed a reduction in cancer cell viability of 46% at 25 μg mL^−1^ and 99% at 50 μg mL^−1^. Zn(CUR)O-A and Zn(CUR)O-B showed 52% and 84% cell viability, respectively, after 48 hours of incubation at the lowest concentration used, while comparable results were observed at higher concentrations, with complete killing of treated cells already at a concentration of 25 μg mL^−1^.

**Fig. 9:**
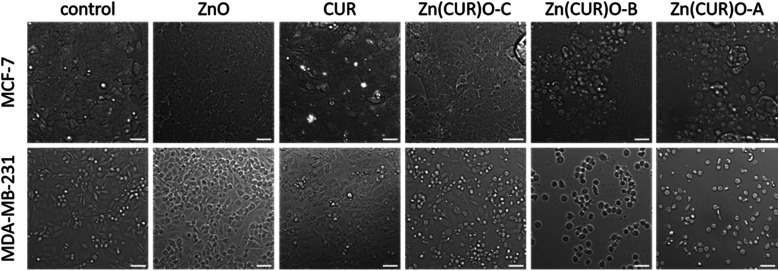
Breast cancer cells were treated with Zn(CUR)O (25 μg mL^−1^ for MCF-7, 12.5 μg mL^−1^ for MDA-MB-231) and compared to both CUR and ZnO NPs. For control, cells were treated with solvent. After 48 h, morphology of the cells was observed under light microscopy. Scale bar = 50 μm.

The findings obtained by the cytotoxicity assay are adequately reflected by microscopy images shown in Fig. 9. Here, cell morphology at 25 μg mL^−1^ and 12 μg mL^−1^ is shown for the MCF-7 and MDA-MB-231 cells, respectively. For both cell lines, control cells as well as ZnO- and CUR-treated cells showed normal morphology and adherent growth. With Zn(CUR)O-C, cells started to alter morphology and detached from the bottom of the cell culture flasks, which are indicators for beginning cytotoxic reaction. Clear signs for cell death can be seen with Zn(CUR)O-B and Zn(CUR)O-A with higher CUR contents in the composites, especially for the MDA cells, where most of the cells display a rounded shape and were completely detached from the bottom.

Overall, a more pronounced effect of the Zn(CUR)O composites was observed against less treatable cancer cells such as MDA-MB-231 compared to the MCF-7 cell line. Such differential responses were also found for pure CUR by Jia *et al.*^60^ who established the involvement of a specific signalling pathway in the mechanism of action of CUR.

**Fig. 10:**
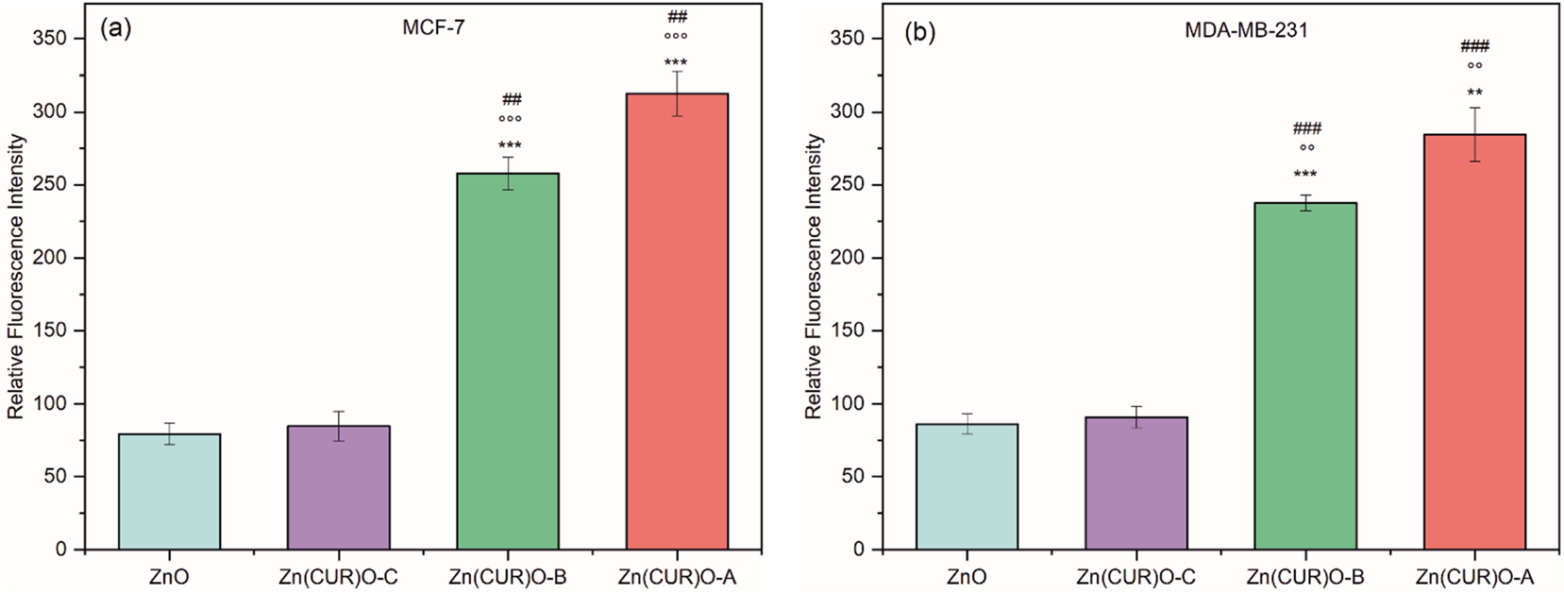
ROS production in breast cancer cells upon 4 h treatment with 50 μg mL^−1^ each of ZnO NPs, CUR and Zn(CUR)O nanocomposites (control treated with solvent was set at 100 relative fluorescence intensity). ***/**/* *vs.* control (DMSO); ^○○○^/^○○^/^○^*vs.* ZnO; ###/##/# *vs.* Zn(CUR)O-C; +++/++/+ *vs.* Zn(CUR)O-B; */°/#/+ *p* < 0.05; **/^○○^/##/++ *p* < 0.01; ***/^○○○^/##/+++ *p* < 0.001.

Comparing the results with the available data in literature^60–64^ regarding the individual anticancer effect of ZnO and CUR towards both MCF-7 and MDA-MB-231, here an improved synergistic effect between ZnO and CUR was demonstrated in the synthesised composite Zn(CUR)O. To explain the toxicity exerted by Zn(CUR)O particles, the hypothesis of induced oxidative stress towards cancer cells has been investigated (Fig. 10). In particular, the production of ROS (*e.g.* O_2_˙^−^, OH^•^, H_2_O_2_) by ZnO and Zn(CUR)O samples was measured by DCFDA technique after 4 h (as per protocol) at the maximum concentration used in viability assays (50 μg mL^−1^). CUR was excluded from these tests, because its own fluorescence emission produced a high signal, which was confirmed by tests performed without treatment of the cells, which showed the same high fluorescence signal. Such problem was already encountered by previous researches.^65^

In the MCF-7 cell line, no increase in ROS was observed for ZnO and Zn(CUR)O-C compared to the control. On the other hand, the results showed a massive production of ROS when cells were treated with Zn(CUR)O-A and Zn(CUR)O-B. Similar results were observed for MDA-MB-231. The evaluation of ROS production showed an overall increase in cell cultures treated with Zn(CUR)O particles with higher concentrations of CUR. This was associated with an overall increased toxicity towards the treated cancer cell lines. Furthermore, such results are consistent with the reported narrowing of band gaps compared to pure ZnO NPs, although further analyses are required to support such a hypothesis.

## 4 Conclusions

We reported experimental evidence that Zn(CUR)O nanocomposite was suitable to be applied as anticancer materials against breast cancer cells. The synthetic procedure involved a straightforward one-pot hydrothermal treatment of Zn(NO_3_)_2_ in the presence of CUR as bioactive molecule. An extensive physicochemical characterization was performed by means of multiple techniques to verify the effect of CUR on the basic properties of ZnO NPs. pXRD proved a progressive broadening of the peaks with increased CUR content, while XPS and SEM analyses revealed a highly inhomogeneous chemical environment around the Zn atoms and ZnO crystals agglomerated in bilobed shaped form instead of the typical hexagonal structure of ZnO, respectively. Diffuse reflection measurements of the composite samples in comparison to the individual compounds revealed a significant decrease in the band gap of the composite material that might also add to the enhanced biological activity. Biological characterisation demonstrated the efficacy of the nanocomposite to induce cell death *via* the induction of oxidative stress, with a synergistic effect between ZnO and CUR.

## Author contributions

Lorenzo Francesco Madeo: conceptualization, investigation, data curation, visualization, writing – original draft. Christine Schirmer: conceptualization, investigation, methodology, data curation, writing – review & editing. Giuseppe Cirillo: conceptualization, methodology, validation, writing – review & editing. Samuel Froeschke: formal analyses, writing – review & editing. Martin Hantusch: investigation, data curation. Manuela Curcio: investigation. Fiore Pasquale Nicoletta: validation, writing – review & editing. Bernd Büchner: supervision. Michael Mertig: resources, funding acquisition, supervision, writing – review & editing. Silke Hampel: conceptualization, resources, funding acquisition, supervision.

## Conflicts of interest

There are no conflicts to declare.

## Supplementary Material
